# A Case Study of Non-specialist Disease Management Using Teladoc Health Support: A Case Report

**DOI:** 10.7759/cureus.60401

**Published:** 2024-05-16

**Authors:** Kenji Numata, Taku Tanaka, Junichi Matsumoto

**Affiliations:** 1 Department of Emergency and Critical Care Medicine, St. Marianna University Hospital, Kawasaki, JPN; 2 Emergency and Disaster Medical Center, Kawasaki Municipal Tama Hospital, Kawasaki, JPN

**Keywords:** emergency service, emergency medicine physician, minor surgeries, emergency medical service, tele health

## Abstract

In Japan, there is a shortage of emergency medicine specialists, often leading non-specialists (physicians who treat conditions outside their area of specialty) to handle cases outside their expertise, which can cause challenges and necessitate specialist support. Starting from December 2023, the St. Marianna University Hospital, which has emergency medicine specialists, began offering overnight emergency outpatient support to Kawasaki Municipal Tama Hospital using the Teladoc HEALTH Mini Cart telemedicine device (Teladoc Health, Inc., CA, USA). The case involved a 44-year-old male with a history of peritonsillar abscess and incisional drainage presented with pharyngeal pain. The treating physician at the Kawasaki Municipal Tama Hospital and a neurologist (the supported physician) examined the patient at 9 PM. An enlarged right tonsil was noted, and a peritonsillar abscess was suspected, prompting a contrast-enhanced CT scan. The results confirmed a 1 cm right peritonsillar abscess. Faced with the decision to transfer the patient to a higher medical facility, the supported physician consulted with the support physician through a Teladoc HEALTH Mini Cart. The St. Marianna University Hospital’s emergency physician (supporting physician) used the Teladoc HEALTH Mini Cart to assess the patient’s overall condition, blood tests, and CT images and advise on antibiotic treatment. A visit to the ear, nose, and throat expert (ENT) the following day was considered sufficient. The supported physician received feedback that the use of the Teladoc HEALTH Mini Cart reduced the burden of nighttime transfers for otolaryngological conditions, which can take several hours. This finding suggests that remote medical support can affect Japan’s emergency medical system.

## Introduction

In Japan, there are insufficient emergency medicine specialists, and even in specialized emergency facilities, emergency physicians are not always on staff. This leads to non-specialist physicians treating conditions outside their specialty (e.g., a cardiologist treating a nosebleed) and sometimes taking these cases [1,2]. Consequently, in areas with a shortage of emergency physicians, there are instances where multiple emergency hospitals refuse to accept patients [3,4].

St. Marianna University Hospital, Kanagawa Prefecture, Japan, is a tertiary hospital with 955 beds. It has 20 emergency medicine specialists, ensuring that emergency physicians are present 24 hrs a day in the emergency outpatient department. Kawasaki Municipal Tama Hospital is a secondary care hospital with 376 beds and has three emergency medicine specialists who are not on duty in the emergency outpatient department around the clock. Consequently, there are issues in dealing with non-specialist diseases. Therefore, from December 2023, remote consultation support using the Teladoc HEALTH Mini Cart telemedicine device (Teladoc Health, Inc., CA, USA) was initiated to assist in difficult decision-making cases. This report presents a case in which the Teladoc HEALTH Mini Cart was used to influence the treatment policy and reduce the burden of care.

## Case presentation

**Figure 1:**
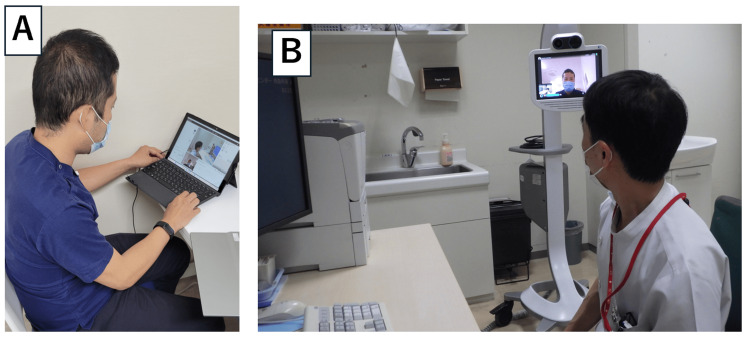
Images of ongoing telemedicine support sessions A. Supporting physician, B. Physician receiving support

**Figure 2:**
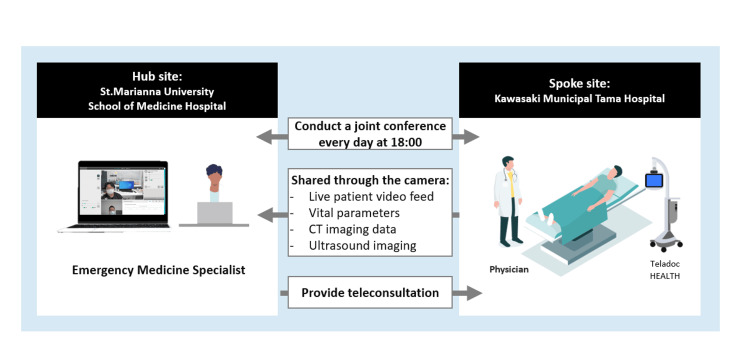
Emergency telemedicine collaboration between Kawasaki Municipal Tama Hospital and St. Marianna University Medical Center The artwork is created by the authors.

The Teladoc HEALTH Mini Cart is a telemedicine device that connects a device (Figure 1) to an application via the Internet to provide near-face-to-face information in real-time [5]. On-call physicians at St. Marianna University Hospital and Kawasaki Municipal Tama Hospital meet via the Teladoc HEALTH Mini Cart at 6 PM. If a decision is difficult for the team at Kawasaki Municipal Tama Hospital, consultation is conducted using the Teladoc HEALTH Mini Cart to determine the course of action (Figure 2).

The physician at the Kawasaki Municipal Tama Hospital who examined the case was a fourth-year postgraduate neurology resident (supported physician). The patient was a 44-year-old male with a history of peritonsillar abscess with prior incision and drainage three years previously presented with pharyngeal pain and walked in at 9 PM. Apart from the fever, his vital signs showed no other notable issues. Blood tests indicated an elevated white blood cell count and C-reactive protein level; no other abnormalities were noted (Table 1).

**Table 1:** Result of Blood test WBC: White blood cells,  RBC: Red blood cells,  Hb: Hemoglobin, PLT: Platelet count, TP: Total protein, Alb: Albumin, UN: Urea nitrogen, Cre: Creatinine, T-Bil: Bilirubin test, AST: Aspartate aminotransferase, ALT: Alanine aminotransferase, CK: Creatine kinase, γGTP: Gamma-glutamyl transpeptidase, Na: Sodium, K: Potassium, Cl: Chloride, PT: Prothrombin time, PT-INR: Prothrombin time-International normalized ratio, aPTT: Activated partial thromboplastin time: CRP: C reactive protein.

Complete blood count		Blood chemistry	
WBC	16000/μl	TP	7.8 g/dl
RBC	4.73×10^12^/l	Alb	3.8 g/dl
Hb	15.1 g/dl	UN	12.7 mg/dl
PLT	33.7 ×10^4^ /μl	Cre	0.72 mg/dl
		T-Bil	0.5 U/
		AST	14 U/L
		ALT	13 U/L
		CK	67 U/L
		γGTP	89 U/L
		Na	137 mEq/L
		K	3.7 mEq/L
		Cl	102 mEq/L
		PT	10.7 sec
		PT-INR	0.89
		aPTT	31.4 sec
		CRP	6.52 mg/dl

**Figure 3:**
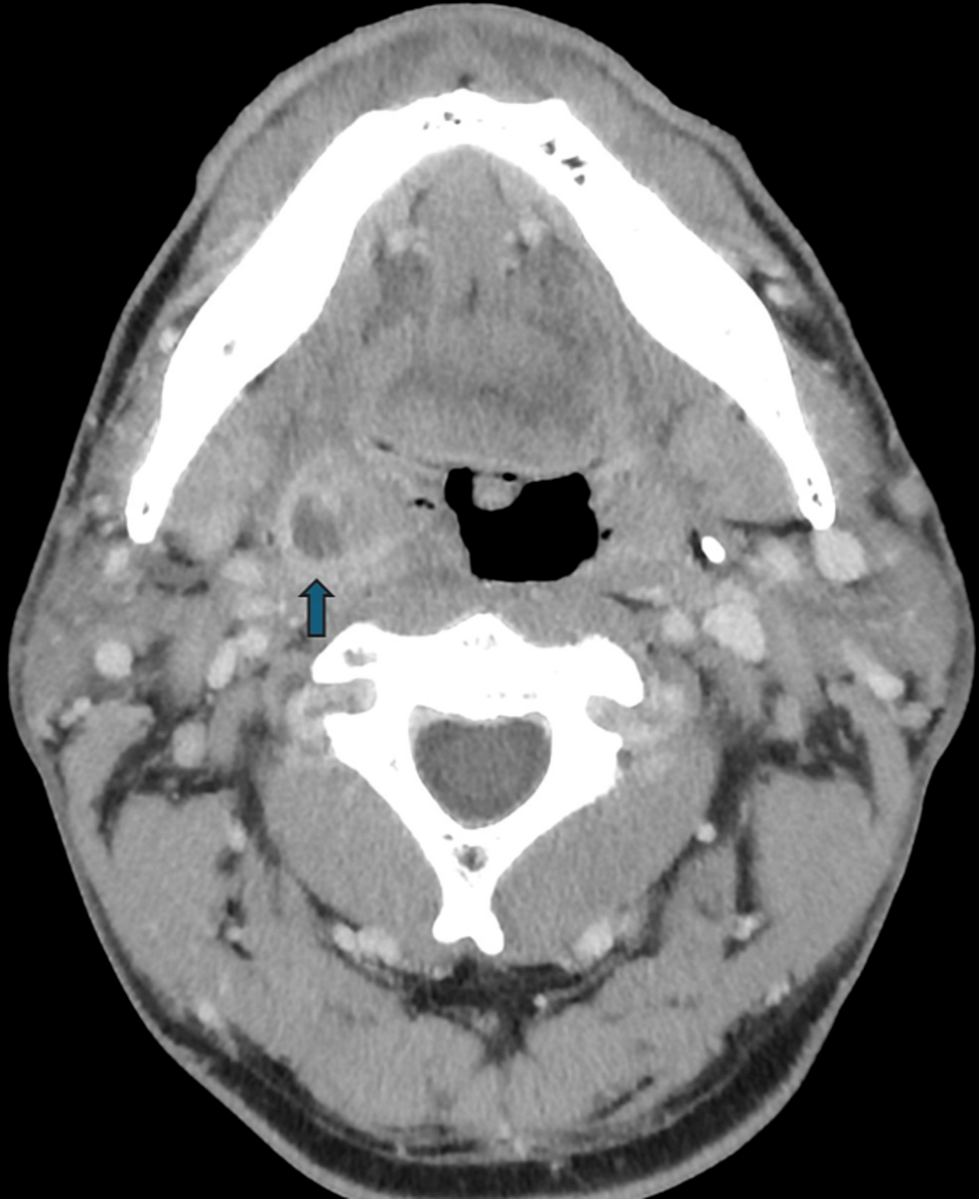
Contrast-enhanced CT image The image shows a right peritonsillar abscess approximately 1 cm in size observed in the right tonsil (blue arrow).

Considering the enlargement of the right tonsil and the history of a peritonsillar abscess with prior incision and drainage, the possibility of a peritonsillar abscess was considered, leading to a contrast-enhanced CT scan. The results confirmed a 1 cm peritonsillar abscess on the right side (Figure 3). As the environment did not permit consultation with an otolaryngologist within the hospital, the supported physician faced uncertainty about whether to transfer the patient to a higher medical facility and consulted with an emergency physician at St. Marianna University Hospital (the supporting physician) through the Teladoc HEALTH Mini Cart device. The supporting physician, using the Teladoc HEALTH Mini Cart, observed the patient’s overall condition, blood tests, and CT images using a camera and discussed them with the support physician. The support physician was concerned about whether to locate and transfer the patient to an otolaryngologist-capable hospital. The supporting physician advised that, given that the airway was clear and the abscess was small, immediate surgical drainage was not required, and antibiotic treatment was recommended with a follow-up at an otolaryngology clinic the next day. The patient was administered ampicillin-sulbactam sodium 3 g intravenously and was scheduled to start taking amoxicillin 500 mg/clavulanic acid 125 mg three times daily from the next day. Following this plan, the patient was continued on antibiotics after visiting the otolaryngology clinic the next day without any surgical intervention. The otolaryngologist suggested that this approach was appropriate and reported no relevant issues.

After using the Teladoc HEALTH Mini Cart, the supported physician was interviewed and responded that the introduction of Teladoc allowed for the efficient management of troubling and potentially urgent cases. The ability to consult a senior physician directly when difficult decisions are made is extremely important. He shared his perspectives in the interview as follows, “Regarding the patients’ treatment time, if we could not have consulted through Teladoc, it would have taken time to find an accepted higher-level medical facility. I believe that Teladoc helped reduce the treatment time by at least three hours.” It should be noted that the mentioned three-hour reduction is based on the physician’s personal assessment rather than a universally applicable figure.

## Discussion

We encountered a case in which remote medical support facilitated decision-making regarding the treatment of a peritonsillar abscess. In Japan, following the COVID-19 pandemic, the use of telemedicine devices for doctor-to-patient interactions has been progressing with scattered reports. However, legislative developments for doctor-to-doctor clinical support have been lagging, especially in emergency outpatient settings [6,7].

The Teladoc HEALTH mini-cart has two main benefits. The first is a reduction in the consultation time between doctors and patients. The supported physician mentioned in an interview that “it could have saved up to three hours.” It should be noted that the mentioned three-hour reduction is based on the physician’s personal assessment rather than a universally applicable figure. Physicians are not always fully versed in managing diseases outside of their specialties. Therefore, when non-specialty diseases are diagnosed, the smooth determination of a treatment plan is not always feasible. Implementing clinical support can make urgent decisions faster, potentially reducing the time doctors and patients spend in medical settings.

The second benefit is the reduction in stress experienced by physicians when examining non-specialty diseases. Owing to the shortage of emergency physicians in Japan, doctors often handle initial treatments for non-specialty diseases, which can be a significant burden [8]. The interviews yielded positive responses, stating that consultations on challenging cases helped finalize decisions. The ability of the Teladoc HEALTH Mini Cart to create an environment similar to that of face-to-face communication is significant. Compared to telephone consultations, which have inherent limitations, they offer an effect comparable to that of in-person interactions [9]. Without the Teladoc HEALTH Mini Cart, consultations relied on traditional calls, suggesting that the Teladoc HEALTH Mini Cart could reduce the barriers and stress associated with consultation for non-specialty diseases. Reports indicate that 18.9% of patient rejections in some regions of Japan are due to non-specialist issues [10]. The introduction of the Teladoc HEALTH Mini Cart may decrease these rejections in the future.

## Conclusions

The authors emphasize the significant benefits of using Teladoc, including enhanced decision-making support for non-specialist physicians and reduced treatment time. This approach minimizes the need for unnecessary transfers and surgical interventions, thereby improving patient care efficiency and reducing the overall burden on healthcare facilities.
